# Sequential modulations of the Simon effect depend on episodic retrieval

**DOI:** 10.3389/fpsyg.2014.00855

**Published:** 2014-08-08

**Authors:** Michiel M. Spapé, Bernhard Hommel

**Affiliations:** Institute for Psychological Research and Leiden Institute for Brain and Cognition, Leiden UniversityLeiden, Netherlands

**Keywords:** Simon effect, cognitive control, action control, episodic retrieval, S-R compatibility

## Abstract

Sequential modulations of conflict effects, like the reduction of the Simon effect after incompatible trials, have been taken to reflect the operation of a proactive control mechanism commonly called conflict monitoring. However, such modulations are often contaminated by episodic effects like priming and stimulus-response feature integration. It has previously been observed that if the episodic representation of a conflicting trial is altered by rotating the stimulus framing 180^∘^ around its axis, the subsequent “conflict adaptation” pattern is eliminated. In Experiment 1, we replicate the findings and provide the basic episodic interpretation. In Experiment 2, we extend the framework to include rotations of 90^∘^, and verify that the episodic effects generalize to scenarios of neutral compatibility. Finally, in Experiment 3, we add complete, 360^∘^ rotations, and show that the episodic manipulation by itself does not eliminate the conflict adaptation patterns – as long as conditions favor episodic retrieval. The experiments are argued to demonstrate that an episodic account of the conflict adaptation effect can most parsimoniously account for the behavioral effects without relying on higher order cognition. Accordingly, we conclude that conflict adaptation can be understood either as critically depending on episodic retrieval, or alternatively reflecting only episodic retrieval itself.

## INTRODUCTION

The time it takes to act is strongly affected by the compatibility between the stimulus and response (Fitts and Seeger, 1953). Simon and Rudell (1967), for example, showed that processing the location of a stimulus automatically triggers a response toward the source of the stimulus, resulting in frequent errors and increased reaction latencies if a stimulus appears in a location opposite to the response. This effect later became known as the *Simon effect* (coined by Hedge and Marsh, 1975), and is one of the more popular effects amongst the range of stimulus-response compatibility phenomena (see for an overview Alluisi and Warm, 1990). Like the Stroop effect (Stroop, 1935), and the flanker-compatibility effect (Eriksen and Eriksen, 1974), the Simon effect follows the general rule that if a task-irrelevant dimension of a stimulus suggests a different response than the relevant dimension, performance will be impaired.

### CONFLICT CONTROL

The location of a stimulus may attract a response in the wrong direction, a word’s meaning sometimes prompts an incorrect verbalization and a peripheral stimulus can distract by cueing inaccurate actions, yet, overall, we are capable of withstanding temptation and can carry out Simon, Stroop, and Eriksen tasks eventually. Models of cognition typically account for this ability by implementing a function which detects and resolves the conflicting responses using executive or conflict control. A conflicting readiness of the motor cortex can indeed be detected using EEG (Stürmer et al., 2002), which might act as a trigger for the cognitive system to utilize cortical areas associated with cognitive control. Then, the control itself could be achieved by facilitating task-related information, thereby supporting the correct response (e.g., Botvinick et al., 1999; Egner and Hirsch, 2005). Alternatively, incorrectly triggered response alternatives could be actively suppressed, similarly biasing the response competition (e.g., Band et al., 2003; Ridderinkhof et al., 2004). Either approach thus assumes that the cognitive system continuously monitors for conflict and enhances control upon detection.

One of the most important sources of evidence for these conflict-control models is provided by the so-called “Gratton Effect,” concerning sequential effects in conflict-inducing tasks (Sheth et al., 2012). This was named after Gratton et al. (1992), who showed that the impact of response-compatible and incompatible flankers on performance is reduced in trials that follow trials with incompatible flankers as compared to trials with compatible flankers. Comparable observations have been made with the Stroop task (e.g., Kerns et al., 2004) and the Simon task (e.g., Praamstra et al., 1999), with either effect being reduced, eliminated and sometimes even reversed after incompatible trials. These observations have been taken as evidence that facing a conflict trial induces an increase of cognitive control, which then proactively facilitates the resolution of conflict in the next trial – resulting in the observed reduction of subsequent conflict effects.

### EVENT FILES

**FIGURE 1:**
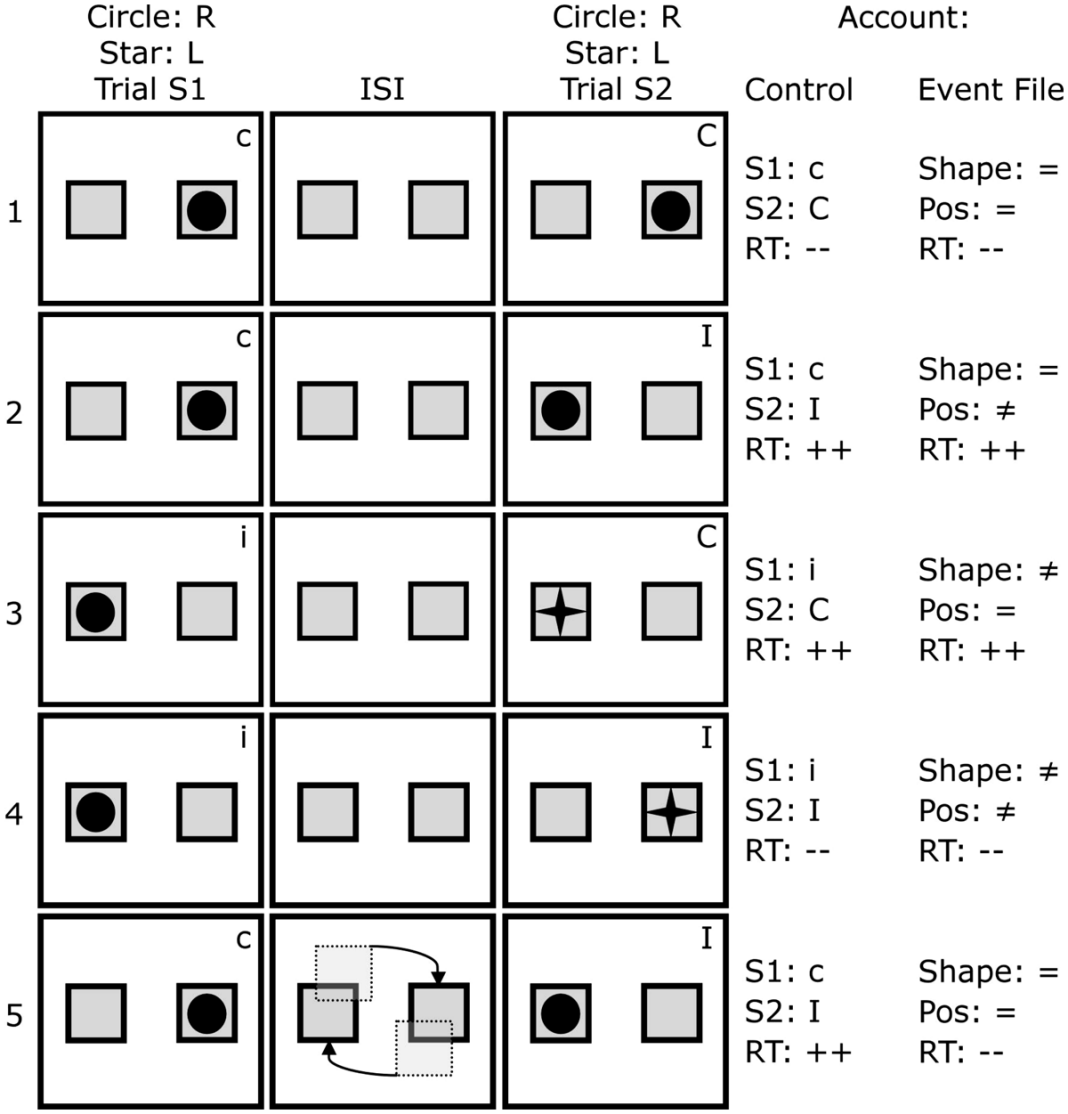
**Sequence of events in five trial pairs and coding in terms of conflict (Control account) and features (Event File account).** Given that the participant responds right (R) to circles and left (L) to stars, the initially compatible (c) trial (S1) in pair 2 is followed by an incompatible (I) trial (S2), which usually results in increased reaction times (RT: ++). Increased reaction times are also predictable in this scenario from an Event File perspective, as the shape is repeated (=) between trials, but not (≠) the position (Pos). This holds for trial pairs 1, 3, and 4 as well. However, divergent predictions were based on the gradual rotation as is depicted in row 5: whereas nothing changes in terms of conflict, the Event File model would predict performance gains (RT: --).

Later considerations and findings have, however, raised some doubts on the interpretation of sequential conflict effects as reflecting a universal, conflict monitoring function. As pointed out by Mayr et al. (2003) and Hommel et al. (2004), sequential relationships between compatibility and incompatibility are naturally confounded with particular patterns of stimulus and response repetitions and alternations. Approximately half a century of research on the effect of priming shows that simply repeating a stimulus or response markedly affects reaction times (Bertelson, 1963; Meyer and Schvaneveldt, 1971) and during sequence modulations, such effects are always present. Given that the combinations of stimulus and response repetitions are not equally distributed across the possible transitions between compatibility conditions, it is possible that at least some sequential modulation effects are due to feature – rather than conflict – repetition (Mayr et al., 2003; see also **Figure 1** row 1).

Sequences of stimulus-response combinations do not only invite priming effects. According to Treisman and colleagues, processing the features of an object leads to a binding of the corresponding feature codes (e.g., Treisman, 1996). They found that the priming effect obtained by repeating an object is enhanced if this object also appears in the same location, suggesting that object features get bound to location codes (Kahneman et al., 1992). Hommel (1998) and Hommel et al. (2001) extended this concept to include action and suggested that object features and action features may be spontaneously integrated into what they call* event files.* If, for instance, a stimulus like a cup of coffee is accompanied by an action like grasping or drinking, the codes of the sensory features (BROWN, WARM, etc.) become integrated with action features (moving the hand toward the object, the typical type of grasp, etc.), resulting in an event file of “drinking coffee.” If one or more features are encountered again the event file is retrieved automatically (cf., Logan, 1988) in a kind of pattern-completion process. This commonly leads to benefits (e.g., if recognition is hampered by suboptimal visibility), but to costs if some features are repeated but now combined with other features. Thus, perceiving the coffee cup again and immediately retrieving the drinking action may lead to an unpleasant surprise if the contents turned cold in the meantime.

These *partial repetition costs* also come into play during sequential conflict studies. Hommel et al. (2004) showed that partial-overlap costs are commonly confounded with the sequence of compatible and incompatible trials in the Simon task (and other conflict tasks). For instance, if a participant responds with *left* to a right-sided stimulus, the event is incompatible (I), whereas responding *left* to a *left* stimulus is compatible (C). Consider how each of these cases could affect subsequent conflict, such as when a *right* response would be required to a *left* location. In terms of the conflict adaptation effect, a compatible trial followed by an incompatible trial (c-I) results in slow reaction times, but an incompatible trial followed by another incompatible trial (i-I) does not. However, if we deconstruct each sequence in terms of their features, it turns out that in the C-I case (e.g., right| right → left| right), one features is repeated, but the other one is not, resulting in partial repetition costs and slow performance (see **Figure 1** row 2). In the I-I case, either the features both change (left| right → right| left, see **Figure 1** row 4), or the features both repeat (left| right → left| right), so there would be no partial repetition costs and faster performance is predicted. Thus, a “conflict adaptation” pattern is elicited by matters entirely unrelated to the change in conflict itself.

### EXCLUDING REPETITIONS IN CONFLICT ADAPTATION

Given that conflict tasks rely on the interrelationship of stimulus and response features, the confound between feature repetitions and the repetitions of compatibility conditions is to some degree unavoidable – at least if the original tasks are left more or less intact. A common workaround solution is to add a layer of complexity to the simple tasks by adding constraints to the randomized design. For instance, some studies have considered only those conditions where no stimulus or response feature is repeated (e.g., Akçay and Hazeltine, 2007) and, given that sequential effects were still obtained, been taken to demonstrate purely executive effects. Even though this approach seems straightforward, it creates two somewhat related problems.

One problem is that excluding any feature overlap between two successive stimuli or stimulus-response episodes does not exclude proactive effects of episodic integration and retrieval (Dutzi and Hommel, 2009). Consider stimuli that vary on two dimensions, such as the visual letters “X” and “O” appearing in red or green. According to the available models of feature integration (e.g., Duncan and Humphreys, 1989), processing a green “X,” for instance, would lead to the competition between codes of the colors GREEN and RED and between codes of the shapes X and O. Collecting sufficient visual evidence should provide sufficient support for GREEN and X, which helps them to outcompete the possible alternatives. Now consider that you process the green X after having seen a red O. If having processed the red O led to the integration of the codes RED and O (Kahneman et al., 1992), they can be assumed to act as a unit and engage in what Duncan (1996) and Duncan et al. (1997) called *integrated competition*. This has advantages for the integrated unit if the stimulus it encodes is repeated but a competitive disadvantage if the stimulus changes: having integrated RED and O makes it easier to reject them as a unit (Duncan and Humphreys, 1989). Any loss of RED in the competition with GREEN will propagate to and thus weaken O as well, and losses of O in the competition with X will propagate to and weaken RED. Empirical evidence for this mechanism has been obtained in search tasks, where non-targets can be more easily rejected if they share features that do not overlap with the target, so that they can be grouped together and rejected as a group (Duncan and Humphreys, 1989). Also in line with expectations from an integrated competition approach is the observation that alternating all features and aspects of a stimulus or stimulus-response episode leads to performance that is as good as (Hommel, 1998) and sometimes even better than performance with complete repetitions (Hommel and Colzato, 2004; Colzato et al., 2008). In any case, it seems clear that avoiding feature overlap between successive trials does not allow one to exclude contributions from feature integration and episodic retrieval.

Another problem in restricting analyses to alternation trials is that this amounts to selecting a single data point which ignores all other interactions between repetitions and alternations of features and leaves out the possibility that control and retrieval might interact, as suggested by Spapé and Hommel (2008). In their study, participants responded to high and low-pitched tones by saying “high” and “low,” while ignoring voices saying “high” and “low.” Unsurprisingly, this created a Stroop-like effect if a word was incompatible with the tone. A typical sequential modulation effect was also obtained, with reduced Stroop effects after incompatible trials. However, if the voice changed between the two successive trials, no such effect remained. Thus, they argued, control information was integrated with the episodic context – i.e., the voice. Only if the episodic context was retrieved did control adaptation affect performance.

Task-switching studies provide support for this interpretation. While switching to a new task is difficult in general (Allport et al., 1994), switching costs are particularly pronounced if the current stimulus was previously encountered in a competing task (e.g., Waszak et al., 2003). This suggests that stimuli and task information are integrated into episodic bindings that are retrieved if the stimulus is encountered another time – which is beneficial if the task has remained the same (as is usual in everyday life) but problematic if the task is different.

Another converging line of evidence comes from task-switching studies that employ a type of conflict task. Evidence from such tasks suggests that a task-switch can result in an elimination of the conflict adaptation effect (Kiesel et al., 2006). In the absence of any similarity between tasks, however, Notebaert and Verguts (2008) observed no sequential effects, suggesting to them that conflict-monitoring acts *locally* (see also Blais et al., 2007).

To summarize, there are reasons to assume that at least some of the effects that are commonly taken to reflect adaptive control actually reflect stimulus-response integration. These effects cannot be avoided by restricting one’s analyses to alternation trials.

### AIM OF STUDY AND RATIONALE

The aim of the present study was to investigate the relationship between adaptive control processes and episodic retrieval in producing sequential modulations in a conflict task, and to test the hypothesis that the former may depend on the latter. To do this, we used an effect that has before been shown to selectively affect episodic retrieval. We replicate previous findings that demonstrate how this simple, episodic manipulation can have strong effects on conflict control. Here, and throughout the article, we provide a side-by-side comparison of the episodic effect in terms of conflict control and feature integration. We then report two additional experiments which confirm separate predictions that relate episodic retrieval to conflict control. In Experiment 2, we show how feature integration effects can be found even if stimuli are repeated in entirely new positions of neutral compatibility. In Experiment 3, we show that the manipulation itself does not eliminate the conflict adaptation pattern by providing evidence that under conditions that favor episodic retrieval, this pattern can be re-established. Accordingly, each experiment concerns the primacy of episodic retrieval: if the conflict adaptation is determined by episodic retrieval, we should conclude it to either require, or be redundant to, episodic retrieval. But let us first consider the episodic effect under consideration.

Already as part of the first studies on feature integration by Kahneman et al. (1992), it has been shown that if a cue is displayed within a bounding box, a priming effect can be observed if a probe appears inside the same box, even if the box has gradually moved between prime and probe to the new position. Thus, feature integration theory allows that the letters and boxes were bound into enduring representations that were updated along with the boxes’ gradual change of position. Simplifying the task considerably, we showed that action features are likewise bound with the object: after a rotation of the stimulus presentation along its axis, a repetition of the action and shape still resulted in performance benefits (Spapé and Hommel, 2010).

Spapé et al. (2011) made use of this effect by transforming the object-reviewing or tracking task into a sequential Simon task. **Figure 1** gives a brief overview of the task and of how the conditions relate to the conflict-control and event-file accounts. If we imagine the task requires a right response for each circle that is portrayed, and a left one for each star, the first row shows a *cI* trial sequence (a compatible initial trial, S1, followed by an Incompatible paired trial, S2) – which typically elicits maximal errors and reaction times. Since the same shape (shape=) is displayed in a new location (pos≠), the event file account likewise predicts poor performance for the same trial sequence. In the second row – and, in fact, in all 8 different combinations of trials (see Hommel et al., 2004) – the same configurations of features cannot distinguish between the two accounts.

However, by gradually having the two boxes exchange positions, as schematically portrayed in the third row, the same predictable effect demonstrated by Spapé and Hommel (2010), should occur. That is, the circle initially displayed right should, following the gradual migration, be represented on the left side, resulting in a complete repetition, which usually results in performance benefits. Meanwhile, there is no reason to assume that the gradual change affects control processes: if registering conflict upon R1 selection suppresses or prevents the processing of stimulus location on S2 presentation, this should not be affected by the presence or absence of a rotation of actually task-irrelevant boxes in between two trials. Therefore, the episodic manipulation of rotating the boxes was not predicted to affect performance from a conflict-control account but only from an event file perspective.

Although neither the boxes, nor the rotation thereof, was relevant to the task or conflict, Spapé et al. (2011) observed strong effects of rotation on conflict adaptation in terms of behavior, as well as event related potentials of the EEG known to be involved in attention and control (the N2) and response readiness (the lateralized readiness potential, LRP): nearly all costs associated with conflict adaptation were removed. However, they did not report the event file analyses, making it difficult to assess whether all predictions from that perspective were fulfilled. Secondly, they also note some curious effects that do not immediately follow from a pure event-file approach. For instance, though they reported that rotation disrupted conflict-adaptation effects, the conflict adaptation effect in terms of psychophysiological indicators did not reverse. That is, in terms of LRP and N2, rotation, the compatibility effect no longer depended on the preceding trial. One could therefore argue that the rotation, rather than causing a change in represented position, induced a *cognitive* *reset,* undoing both the conflict adaptation *and* the event file.

In order to better understand the effects of rotation on both conflict control and event files, we report in this series of experiments both types of analysis side-by-side. In the *conflict-control* analysis, we examined the data in terms of the sequence of compatibility conditions, testing whether S1 incompatibility would reduce the S2 compatibility effect, and aiming to replicate that rotation, a factor that should be meaningless from a conflict-control point of view, affected the interaction between S1 and S2 compatibility. In the *event-file* analysis, we examined whether response-repetition and stimulus-location would interact in the standard partial repetition-cost pattern: complete repetitions and alternations should result in better performance than repeating one feature, but not the other (Hommel and Colzato, 2004). Of particular theoretical interest here was whether rotation would tend to eliminate these effects (as the two-event-files account of Spapé and Hommel, 2010, would suggest) or even reverse their sign (as a one-file extension of the approach of Kahneman et al., 1992, might imply).

In Experiment 1, we will first demonstrate these two types of analyses in a straight replication study. In the subsequent two experiments, we continue this pattern but extend it to new territories. In Experiment 2, we show the effects of rotating conflicting objects to completely new (in event file terms) and sometimes neutral (in conflict terms) locations by including rotations of 90^∘^. Finally, in Experiment 3, we allow rotations to return to their initial position as well, using a full 360^∘^ rotation which completes the picture and confirms that the rotation by itself does not destroy the conflict adaptation effect.

## EXPERIMENT 1

In a Simon task, participants respond to a non-spatial stimulus feature by carrying out a left or right response, whilst ignoring the irrelevant location of the stimulus. In our version, participants responded to circles and stars by pressing a left or right key. The stimuli appeared in the left or right of two constantly visible boxes. Trials were presented in pairs, so that one circle or star was presented (S1) and responded to (R1) before a second circle or star (S2) appeared to signal a second response (R2). The boxes remained visible in between the two trials of a pair and were rotated by 180^∘^ in 50% of the trials.

### METHOD

#### Participants

Eighteen students from Leiden University voluntarily participated in this experiment in exchange for money or course credits.

#### Apparatus and stimuli

Stimuli were presented on a flat-screen 17^′′^ CRT monitor in 800 × 600 pixels resolution and a refresh-rate of 120 Hz. A Pentium-IV dual 1.67 GHz PC running E-Prime (1.1, SP3) on Windows XP SP2 controlled stimulus-presentation and recorded reactions via the USB connected keyboard. The boxes were gray (RGB value of 128, 128, 128), black-lined squares of 60 × 60 pixels or approximately 32 × 32 mm presented against a silver (RGB value of 191, 191, 191) background. The target itself was also 60 × 60 and was either a circle or a four-pointed star. Boxes were presented 180 pixels (approximately 96 mm) left and right from the center of the screen and also kept at this distance during the gradual shifts in location.

#### Procedure

**FIGURE 2:**
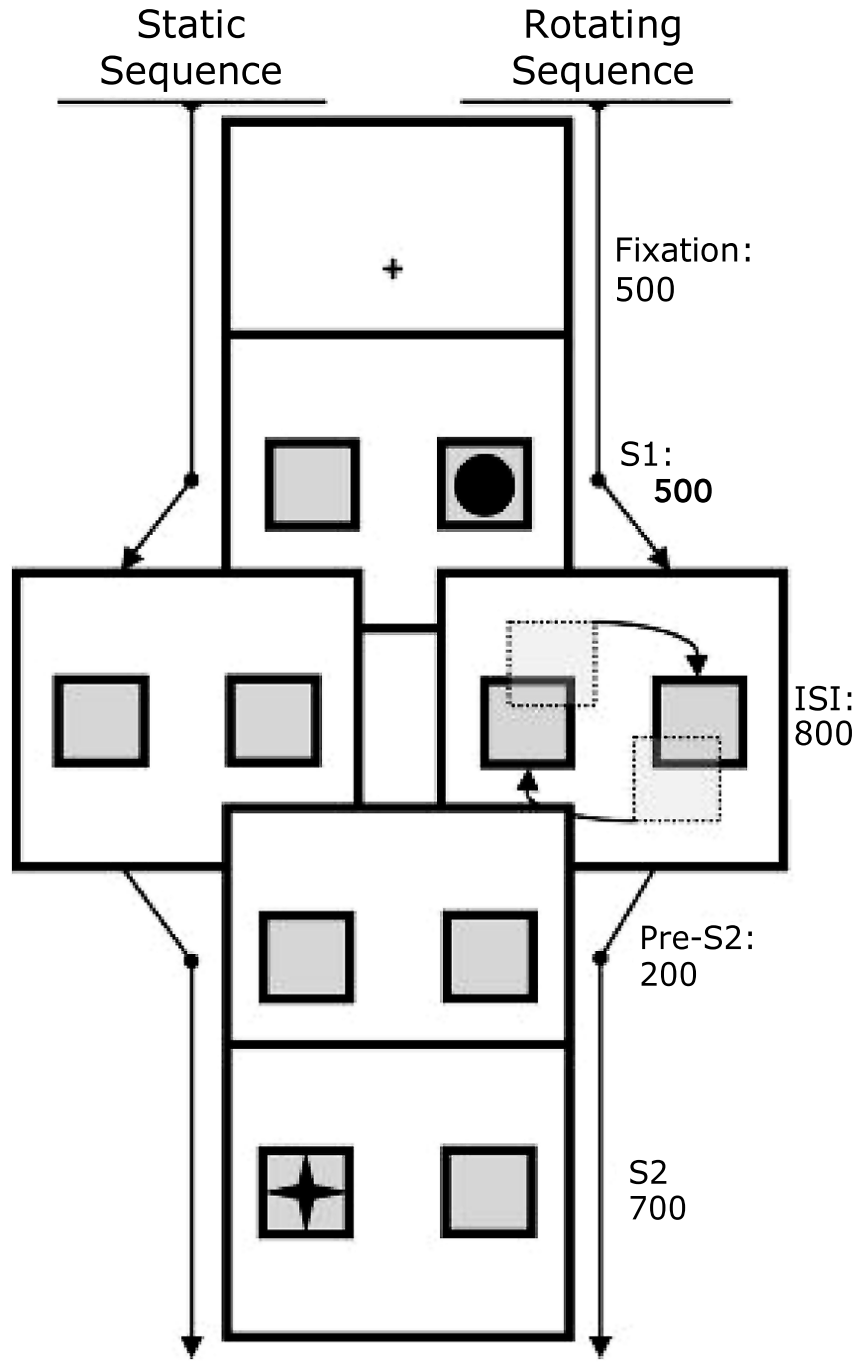
**Schematic depiction of the trial-sequence of two example trials.** After presenting a fixation crosshair, two boxes were presented for 500 ms in the left and right of the screen, one containing the shape (S1) to which participants were required to respond. In the “static” condition (left), an inter-stimulus interval (ISI) followed in which the boxes stood still for 800 ms, whereas in the rotating condition, they rotated around their axis during this ISI. In both conditions, the boxes were statically presented for another 200 ms before the second target (S2) was shown. S2 was shown for 700 ms before an inter-trial interval of 1100 ms ended the trial.

As outlined in **Figure 2**, a fixation cross was presented for 500 ms, after which the two boxes were presented on the left and right of the screen, one of them containing the target shape (S1) to which participants were required to respond. After 500 ms, the targets were no longer shown on the screen. In the “static” condition, the boxes stood still, without targets, for 800 ms, whereas in the rotating condition, they rotated around their axis at a speed of approximately 4^∘^ with each 44 ms. After the 800 ms, both in the static condition and the rotating condition, the boxes were presented for another 200 ms before the second target (S2) was presented. S2 was shown for 700 ms before a screen with feedback informed the participant of the performance. This last screen also comprised the inter-trial interval and was shown for 1100 ms.

Following the instruction, the first 20 trials of the experiment were considered practice of acquiring the mapping between circles or stars with a <Q> or <P> keypress. Half of the participants were to press <Q> for stars and <P> for circles, the other half received the opposite stimulus-response mapping. They were required to respond as quickly and accurately as possible and were shown a personal score next to a high score which they were encouraged to break. Points were awarded for responding fast (1 point for each reaction below 600 ms) and accurately (1 point for each accurate reaction). Although there was no monetary or other incentive for breaking the high score, most participants did indicate being positively motivated to aim to break the (fictional, computed as 3 × number of trials) high score. The experimented lasted about 30 min.

#### Design

Results were coded so as to analyze them with two different three-factor repeated measures designs. First, predictions from the conflict adaptation approach were tested by considering rotation (static versus rotating), compatibility of the first stimulus and response, and compatibility of the second stimulus and response. Second, predictions from the event coding approach were tested by considering rotation, shape/response repetition, and stimulus-location repetition. For both types of analyses, the eight design cells resulting from crossing these factors were replicated an even 32 times. The four blocks consisted of the 64 possible, randomly presented, combinations of rotation (versus non-rotation), direction of rotation (clockwise versus counter-clockwise), target shapes (in S1 and S2) and the location of the targets (in S1 and S2).

### RESULTS

Responses (in this as well as Experiments 2 and 3) with latencies longer than 1000 ms were not considered (S1: 2.0%, S2: 1.2%), and all incorrect reactions to S1 or S2 were excluded from RT analyses. Few errors were made during S1 (*M* = 6.7%, SD = 5.7%) and S2| S1 (*M* = 4.3%, SD = 2.7%, S2 errors given correct S1).

#### Conflict-control analysis

In a repeated measures analysis of variance on the RT to S2 with rotation, S1 compatibility and S2 compatibility as factors, reactions were some 30 ms faster after seeing the boxes rotate, *F*(1,17) = 52.09, MSE = 31661.46, *p* < 0.001, while error rates were not affected, *F*(1,17) = 2.03, MSE = 0.01, *p* > 0.1. Compatibility on S1 had no effect on RT, *F*(1,17) = 0.08, MSE = 34.73, *p* > 0.7, but increased accuracy, *F*(1,17) = 4.54, MSE = 0.01, *p* < 0.05. Participants were 30 ms slower if S2 and R2 were incompatible, *F*(1,17) = 73.05, MSE = 32753.76, *p* < 0.001, and made 4% more errors, *F*(1,17) = 24.784, MSE = 0.06, *p* < 0.001. The conflict-adaptation-type effect was replicated, as evidenced by a significant interaction between S1 compatibility and S2 compatibility on RT, *F*(1,17) = 25.49, MSE = 16578.71, *p* < 0.001, and errors, *F*(1,17) = 21.11, MSE = 0.02, *p* < 0.001: the compatibility effect was larger after compatible than after incompatible trials. Indeed, on static trials, the Simon effect was significantly *inverted* after incompatible trials, *t*(17) = 2.27, *p* < 0.04, although not for errors, *t*(17) = 0.29, *p* > 0.7. However, this effect was modulated by rotation in both RTs, *F*(1,17) = 40.93, MSE = 14174.49, *p* < 0.001, and errors, *F*(1,17) = 12.63, MSE = 0.02, *p* < 0.005. To test the effect of conflict-adaptation under static and rotating conditions, two *t*-tests of the interactions between S1 and S2 compatibility were computed. Whereas the interaction between S1 compatibility and S2 compatibility was very pronounced and reliable with static boxes, RTs: *t*(17) = 6.17, *p* < 0.001, errors: *t*(17) = 4.72, *p* < 0.001, it entirely disappeared with rotating boxes, RTs: *t*(17) = 0.50, *p* > 0.6, errors: *t*(17) = 0.59, *p* > 0.5, see **Table 1A**.

**Table 1A:** Experiment 1, compatibility and conflict-adaptation results.

					Compatibility effect		Conflict
S1	Compatible (c)		Incompatible (i)		After c	After i	Adaptation
S2	C	I	C	I	cI – cC	iI – iC	(cI – cC) – (iI – iC)
**Reaction times**
Static	448 (9)	515 (15)	489 (13)	473 (11)	66	-16	83
Rotating	432 (10)	469 (12)	436 (12)	470 (11)	37	34	3
**Error rates**
Static	2 (1)	11 (2)	4 (1)	3 (1)	9	0	10
Rotating	2 (1)	6 (1)	2 (1)	5 (1)	4	3	1

*Reaction times, error rates and standard errors (in parentheses) for S2 (the probe or “current trial”) as a function of S1 compatibility, S2 compatibility, and rotation. Effect sizes to the right show the compatibility (Simon) effect and how it is affected by preceding (S1) compatibility. The conflict-adaptation effect is measured as the degree to which the compatibility-effect of S2 is attenuated after incompatible S1s.*

#### Event-file analysis

**FIGURE 3:**
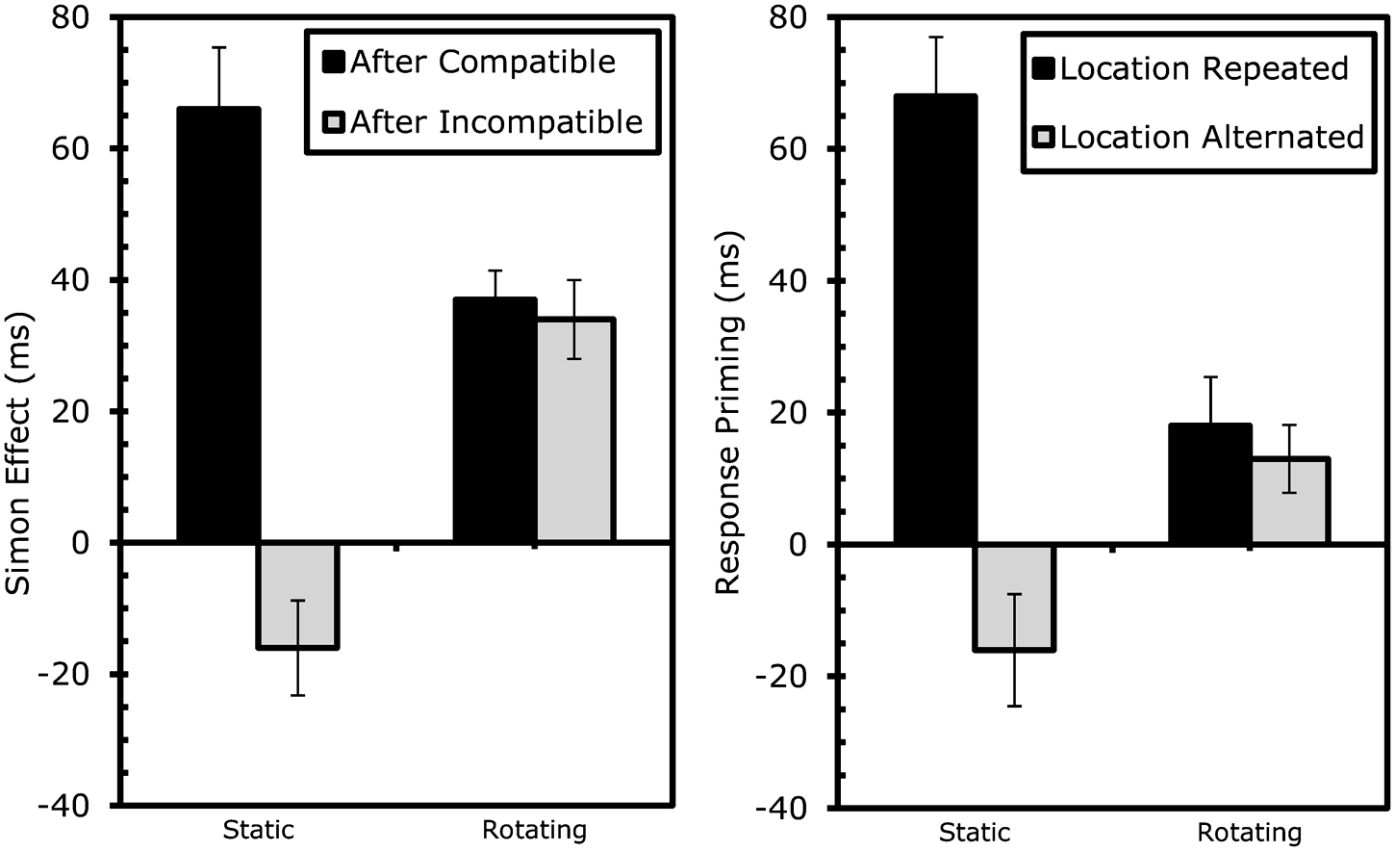
**Results Experiment 1: effects of rotation on sequential Simon effects (left) and event-coding (right).** Conflict-adaptation was measured as the reduction in Simon effect after incompatibility. Event-coding effects were measured as the decrease in response-priming benefits if the location did not repeat along with the required response. Error bars represent standard error of the Simon (left) or response-priming (right) effect within the specific condition.

Rotation had a comparable effects here, both on RTs, *F*(1,17) = 52.39, MSe = 32831.63, *p* < 0.001, and errors, *F*(1,17) = 2.23, MSe = 0.01, *p* > 0.2. The only other main effect indicated that responses were faster if the shape/response was repeated, *F*(1,17) = 18.77, MSe = 15887.34, *p* < 0.001. As expected (Hommel et al., 2004), stimulus-location repetition interacted significantly with shape/response repetition in RTs, *F*(1,17) = 25.34, MSe = 17916.27, *p* < 0.001, and errors, *F*(1,17) = 23.84, MSe = 0.03, *p* < 0.001. The standard cross-over interaction indicated that performance was better with complete repetitions and alternations than with partial-repetitions (see **Table 1B**). In other words, performance was good if stimulus shape, stimulus location, and the response was repeated or if all three features changed, but comparatively bad if shape and response were repeated while stimulus location alternated or if shape and response alternated while stimulus location repeated. This interaction was further modified by rotation in both RTs, *F*(1,17) = 43.47, MSe = 14077.43, *p* < 0.001, and errors, *F*(1,17) = 12.83, MSe = 0.02, *p* < 0.005. As shown in **Table 1B** and **Figure 3**, partial-repetition costs and, thus, the interaction of location and shape/response repetition) were restricted to static boxes and disappeared with rotating boxes. Interestingly, overlap costs were not negative in the rotation condition.

**Table 1B:** Experiment 1, response-priming and Event-file results.

	Location				Response priming		Partia
	Repeated (R)		Alternated (A)		Loc. R	Loc. A	Overlap-costs
Response	R	A	R	A	RA – RR	AA – AR	(RA-RR) – (AA-AR)
**Reaction times**
Static	451 (9)	519 (15)	487 (14)	470 (12)	68	-16	84
Rotating	441 (7)	460 (13)	446 (10)	459 (13)	18	13	5
**Error rates**
Static	2 (1)	8 (2)	7 (2)	3 (1)	5	-5	10
Rotating	3 (1)	4 (1)	4 (1)	4 (1)	1	0	0

*Reaction times, error rates and standard errors (in parentheses) for responses to S2 (the probe or “current trial”) as a function of rotation, S1-S2 location- and response-repetition. Effect sizes to the right show response-priming effects and how they are modulated by location-repetition. Partial Overlap-costs (the effect of repeating only the location or response between S1 and S2) were measured as the difference between response-priming effects of location-repetitions (Loc. R) and location-alternations (Loc. A).*

### DISCUSSION

The outcome of Experiment 1 is rather clear-cut. With static boxes, we replicated the earlier finding that the Simon effect is larger after compatible than after incompatible trials (Stürmer et al., 2002). As explained in the introduction, it has often been suggested (cf. Stürmer et al., 2002) that Simon stimuli are processed via two pathways, with the detection of a conflict triggering the gating or blocking of the automatic route (Botvinick et al., 1999). This account would predict less or no Simon effect after incompatible trials: after encountering an incompatible trial, the automatic location-to-response route becomes to some extent tagged as uninformative. However, this account cannot easily explain the *reverse* Simon effect (compatible trials with slower responses) observed after incompatible trials as observed here (and by others, e.g., Hommel et al., 2004). Alternatively, the automatic route may be actively suppressed (e.g., Ridderinkhof, 2002), so that after incompatible trials, it becomes harder to follow any route that coincides with the response that is suggested by the automatic route. This model can therefore account for reduced performance for compatible trials after incompatible trials, resulting in the reverse Simon effect observed here. In any case, our findings for static conditions are consistent with at least some versions of the conflict-control approach.

The rotation conditions show a close replication of Spapé et al. (2011) in terms of their strong effect on the conflict adaptation effect, which remains harder to understand from a conflict-control perspective. Since the boxes themselves are unlikely to cause any conflict, and as they do not even contain any targets while being rotated, there is no reason why moving the boxes should have any effect on conflict-adaptation. Accordingly, we see no way how conflict-monitoring theories can account for our observations. Would the sequential effects have been only smaller (but not absent) in the rotation condition, one could have argued that they consist of an adaptation component and an independently operating episodic component – with the latter being eliminated and the former being constant. Given the total elimination of the effect, however, this does not seem to be an option.

From an event-file perspective (Hommel et al., 2001; Hommel, 2004) the outcome pattern makes more sense. As predicted, rotating the boxes strongly affected the interactions between stimulus- and response-repetition effects. To the degree that these interactions reflect the creation and later retrieval of feature bindings, this suggests that rotation at least co-determined how the features were coded. There are two hypotheses of how the gradual rotation affects the feature representation. The first would be an extension of Kahneman et al.’s (1992) logic to event files that contain response information and holds that R2 performance is affected by the retrieval of one event file only. In particular, it assumes that rotating the boxes leads to an update of the event file that had just been formed to represent the S1-R1 episode: left stimulus codes are turned into right and right stimulus codes into left codes. If so, the event-file analysis should show regular partial-overlap costs under static conditions, but “negative costs” under rotation conditions, indicating a performance gain if, for instance, the same response is required in the new, updated location.

However, given that this was clearly not the case, the findings may be better understood in terms of our previous suggestion of a two-event-file account (Spapé and Hommel, 2010). This second hypothesis assumes that R2 performance under rotation conditions is affected by two event files simultaneously: one that codes the original S1-R1 episode and another that represents the post-rotation state of affairs. Given that the spatial stimulus codes in these two files are always inconsistent; their effects will tend to cancel each other out. If so, one would expect positive partial-overlap costs under static conditions costs but no overlap costs after rotation. This is exactly the pattern we have obtained, suggesting that the two-file account is more realistic.

To summarize, Experiment 1 clearly replicates Spapé et al. (2011), providing evidence that, at least under the conditions tested here, sequential modulations of Simon effects are entirely due to episodic binding and retrieval. There is one fly in the ointment, however: Although the null effect of event coding in the rotation condition may result from the counteracting effects from two event files, we have no direct evidence that it does. Rather than creating a second, updated file when the boxes move, the cognitive system may simply erase the previous (or any) file whenever a movement or any other dramatic change of the visual display is encountered (Zacks et al., 2007). In the next experiment, we therefore aimed for positive evidence that event files are actually updated and that the updated files affect performance.

## EXPERIMENT 2

In Experiment 2 we also rotated the boxes in which stimuli appeared in between S1 and S2 presentation. Two of the three rotation conditions conceptually replicated Experiment 1: A 0^∘^ rotation condition corresponded to the static condition of Experiment 1, in which the boxes were not moving, and a 180^∘^ rotation condition corresponded to the rotation condition of Experiment 1. The outcomes of these two conditions were expected to replicate the basic finding that conflict-adaptation-type effect should be restricted to the 0^∘^ condition and be eliminated in the 180^∘^ rotation condition. The more important manipulation, however, was the introduction of a third rotation condition. Here, the boxes were rotated only 90^∘^, so that boxes did not overlap between S1 and S2 displays. S2 could still appear either in the same box (e.g., in the location toward which the box where S1 appeared was rotated) or in the other box (i.e., in the location opposite to the box where S1 appeared was rotated). However, given that the 90^∘^ rotation always moved the boxes to locations that were not occupied by the boxes in the S1 display, old and new event files could no longer cancel each other out. Accordingly, their effects should be reliable and more pronounced than in the 180^∘^ condition.

### METHOD

Twenty-two students from Leiden University between the age of 19 and 25 took part in the study in exchange for money or course credits. The procedure was the same as in Experiment 1, except that S1 and S2 could also appear above and below the screen center, that the boxes could be rotated by 0, 90, or 180^∘^, and that the rotation could take 800 or 1200 ms (a factor that was introduced for reasons unrelated to the present study and that therefore was not considered further in the analyses). The two boxes could thus be either horizontally or vertically oriented in both the S1 and S2 displays, which created four types of transition: horizontal-to-horizontal (H–H) and vertical-to-vertical (V–V), the two 180^∘^ conditions, and horizontal-to-vertical (H–V) and vertical-to-horizontal (V–H), the two 90^∘^ conditions. The experiment lasted about 40 min.

### RESULTS

Trials with incorrect responses to S1 (11.6%) were excluded from the error analyses, and trials with incorrect responses to S1 or S2 (another 11.6%) were excluded from RT analyses.

#### Conflict-control analysis

**FIGURE 4:**
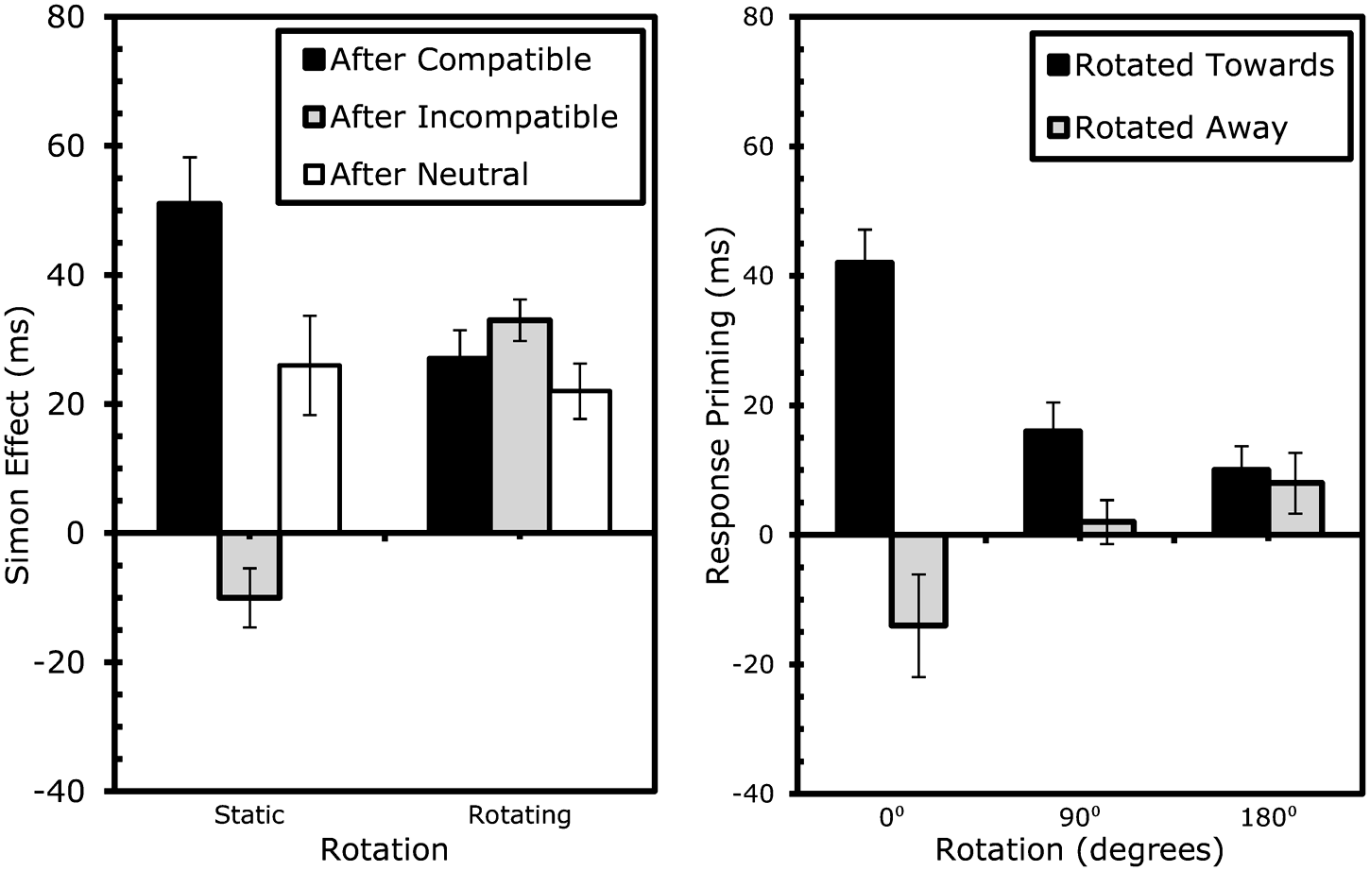
**Results Experiment 2: effects of rotation on sequential Simon effects (left) and event-coding (right).** Error bars represent standard error of the Simon (left) or response-priming (right) effect within the specific condition.

The factors were again rotation (rotated vs. static) and compatibility of S1 and S2 (compatible vs. neutral vs. incompatible), where the compatible and incompatible conditions were taken from the horizontal displays and the neutral conditions from the vertical displays. In repeated measures ANOVAs, S1 compatibility approached significance in RTs, *F*(2,42) = 3.01, MSe = 575.99, *p* < 0.07, but not in error proportions, *F*(2,42) = 0.79, MSe = 0.002, *p* > 0.7; while S2 compatibility had a significant effect on both RTs, *F*(2,42) = 42.82, MSe = 20235.00, *p* < 0.001, and errors, *F*(2,42) = 48.85, MSe = 0.21, *p* < 0.001. Participants were 15 ms faster with rotating trials, *F*(1,21) = 26.19, MSe = 13974.68, *p* < 0.001, but not more often correct, *F*(1,21) = 1.75, MSe = 0.01, *p* > 0.2. Rotation modulated the effect of S1 compatibility, *F*(2,42) = 4.55, MSe = 626.31, *p* < 0.02, for RTs, but not errors, *F*(2,42) = 0.46, MSe = 0.001, *p* > 0.6. The standard conflict-adaptation pattern was found for RTs, *F*(4,84) = 10.54, MSe = 2521.80, *p* < 0.001, and errors, *F*(4,84) = 8.60, MSe = 0.03, *p* < 0.001, with larger S2 compatibility effects after compatible than incompatible S1 (effect sizes: 39 ms and 13% as opposed to 12 ms and 2% respectively). As can be seen in **Table 2A** and **Figure 4**, adaptation-type patterns after a neutral S1 were in between (24 ms, 7%). The three-way interaction was also significant in RTs, *F*(4,72) = 14.65, MSe = 3527.93, *p* < 0.001, again showing that rotation eliminated all adaptation-type effects: strong conflict-adaptation was found under static conditions, RTs: *t*(21) = 5.57, *p* < 0.001, errors: *t*(21) = 4.59, *p* < 0.001, but insignificant under rotating conditions, RTs: *t*(21) = 1.10, *p* > 0.1, errors: *t*(21) = 0.15, *p* > 0.8.

**Table 2A:** Experiment 2, compatibility and conflict-adaptation results.

		S2			Conflict Adaptation	
Rotation	S1	Compatible	Incompatible	Neutral	C – I	(cI-cC) – (iI-iC)
**Reaction times**
Static	Compatible	378 (4)	428 (9)	410 (6)	51	
	Incompatible	415 (9)	405 (7)	418 (8)	-10	61
	Neutral	402 (6)	428 (7)	406 (6)	26	
Rotating	Compatible	386 (5)	413 (6)	396 (6)	27	
	Incompatible	379 (5)	412 (6)	398 (6)	33	-6
	Neutral	389 (5)	411 (6)	399 (6)	22	
**Error rates**
Static	Compatible	2 (1)	19 (3)	6 (2)	17	
	Incompatible	10 (2)	6 (2)	5 (1)	-4	21
	Neutral	3 (1)	11 (2)	8 (1)	7	
Rotating	Compatible	3 (1)	12 (2)	6 (1)	*6pt9	
	Incompatible	4 (1)	12 (2)	5 (1)	8	0
	Neutral	4 (1)	11 (1)	5 (1)	8	

*Reaction times, error rates and standard errors (in parentheses) for responses to S2 as a function of S2 compatibility, S1 compatibility, and rotation. Effect sizes to the right show how the compatibility effect is affected by preceding (S1) compatibility (see ??). Note that Neutral S1s indicate trials in which the stimuli were vertically aligned.*

#### Event-file analysis

To establish whether we were able to replicate our findings of Experiment 1, we conducted repeated measures ANOVAs with rotation (rotated vs. static), location-repetition and response-repetition on the conditions where the displays were horizontally aligned and rotated either 180^∘^ or not at all. Rotation had a significant effect on RTs, *F*(1,21) = 19.65, MSe = 7459.94, *p* < 0.001, and a marginally significant effect on errors, *F*(1,21) = 4.19, MSe = 0.03, *p* < 0.06, with rotated conditions being 13 ms faster and 2.7% more often correct. Location repetitions were slightly (7 ms) slower, *F*(1,21) = 12.33, MSe = 2404.12, *p* < 0.005, but not less often accurate, *p* > 0.6, than location alternations, whereas response repetitions were significantly faster (10 ms), *F*(1,21) = 5.81, MSe = 4536.24, *p* < 0.03, but not more often accurate, *p* > 0.2, than response alternations. In a similar fashion to Experiment 1, response-repetition interacted significantly with location-repetition for both RTs, *F*(1,21) = 42.02, MSe = 13192.65, *p* < 0.001, and errors, *F*(1,21) = 24.17, MSe = 0.24, *p* < 0.001, the effect of which itself was modulated by rotation for RTs, *F*(1,21) = 29.89, MSe = 13046.56, *p* < 0.001, and errors, *F*(1,21) = 16.23, MSe = 0.16, *p* < 0.001. These findings replicate our observations in Experiment 1 and confirm that they represent a robust pattern.

The next analysis compared the two rotation conditions, which required us to recode the data. We compared trials where S1 location (i.e., the box that contained S1) was *rotated toward* the location of the upcoming S2 (e.g., if S1 appeared in the top box, this box was rotated to the right, and S2 appeared in the right box) with trials where the box holding S1 was *rotated away* from the location where S2 would appear (e.g., if S1 appeared in the top box, this box was rotated to the right, and S2 appeared in the left box). ANOVAs were run with the factors shape/response repetition (repetition vs. alternation), direction of rotation (toward vs. away the location of S2), and degree of rotation (0^∘^ vs. 90^∘^ – taken from V–H and H–V transitions – vs. 180^∘^ – taken from V–V and H–H transitions). Repeated responses were faster, *F*(1,21) = 7.28, MSe = 3347.00, *p* < 0.02, but not more accurate, *F*(1,21) = 0.001, MSe = 0.00, *p* > 0.9. Direction of rotation had no effect on RT, *F*(1,21) = 0.09, MSe = 9.83, *p* > 0.7 or errors, *F*(1,21) = 0.01, MSe = 0.00, *p* > 0.9. Degrees of rotation had no effect on RT, *F*(1,21) = 0.10, MSe = 9.09, *p* > 0.7, but had a small effect on errors, *F*(1,21) = 6.32, MSe = 0.00, *p* < 0.03, with 90^∘^ rotations eliciting 1.0% more errors than rotations of 180^∘^. More importantly, shape/response repetition significantly interacted with direction of rotation in RTs, *F*(1,21) = 6.76, MSe = 752.28, *p* < 0.02, and marginally in errors, *F*(1,21) = 3.13, MSe = 0.00, *p* < 0.09. While rotations toward the target location generally resulted in (4 ms) faster, more (0.7%) accurate reactions than with rotations away with repeated shapes/responses, rotations away yielded (5 ms) faster, more (0.6%) accurate responses than rotations toward with alternated shapes/responses. This effect itself, however, was modulated by the degrees of rotation, for both RTs, *F*(1,21) = 7.82, MSe = 436.06, *p* < 0.02, and errors, *F*(1,21) = 8.57, MSe = 0.01, *p* < 0.01. *Post hoc* tests comparing the partial-repetition costs (see **Table 2B** for calculus) for the 90^∘^ and 180^∘^ revealed that partial-repetition costs were larger in the 90 than in the 180^∘^ condition, for both RTs, *t*(21) = 2.80, *p* < 0.02, and errors, *t*(21) = 2.93, *p* < 0.01 (see **Table 2B** and **Figure 4**).

**Table 2B:** Experiment 2, response-priming and Event-file results.

	Location / Rotation				Response Priming		Partial
	Toward (R)		Away (A)		Loc. R	Loc. A	Repetition costs
Response	R	A	R	A	RA – RR	AA – AR	(RA-RR) – (AA-AR)
**Degrees**	**Reaction times (ms)**	
0^∘^	385 (5)	427 (8)	403 (7)	389 (8)	42	-14	56
90^∘^	391 (5)	407 (7)	398 (6)	399 (6)	16	2	15
180^∘^	394 (5)	403 (6)	394 (5)	402 (7)	10	8	2
**Degrees**	**Error rates (%)**	
0^∘^	2 (1)	20 (3)	10 (2)	4 (1)	18	-6	24
90^∘^	5 (1)	8 (1)	7 (1)	6 (1)	3	-1	4
180^∘^	7 (1)	5 (1)	6 (1)	5 (1)	-2	0	-2

*Reaction times, error rates and standard errors (in parentheses) for S2 as a function of degrees of rotation (0^∘^ indicating static conditions), response-repetition and (rotated) location. Note that, different from ??, rotating is either “toward” – as with conditions where the box containing the stimulus in S1 gradually rotated toward the location in which S2 was presented – or “away” – under conditions in which the box containing S1 rotated away from the location in which S2 was presented. Thus, with rotations of 0^∘^, rotating toward and away are tantamount to location-repetitions and alternations respectively.*

### DISCUSSION

The findings of Experiment 2 demonstrate that the degree of rotation matters and that, as expected, the 90^∘^ rotation condition produces stronger binding effects. The results of Experiment 1 showed that after rotating the stimulus display for 180^∘^, both conflict-adaptation and partial-repetition costs were reduced to numbers around zero. This was explained as either the result of rotation resulting in two-event-files, or it effectively removing the (memory of the) previous event. Experiment 2 shows that after a 90^∘^ rotation, in which S2 appeared at a new location, partial repetition costs increase once again to levels clearly above 0, demonstrating clear episodic effects even after the rotation.

One might argue, however, that the results of Experiment 2, merely show that rotation in and of itself reduces feature-integration, and/or conflict-adaptation. The results of both Experiments 1 and 2 suggested that this may be so, since partial-repetition costs were found to be smaller with each ‘extra degree of rotation’: from a sizable 80 ms in 0^∘^ (i.e., static) conditions, via a smaller but significant 15 ms in 90^∘^ conditions to insignificant near-zero in 180^∘^ conditions. Thus, one could simply argue that the more the boxes rotate, the lesser be the binding. Likewise, rotation itself could have disrupted conflict adaptation, as after rotating the boxes, no conflict-adaptation was found. If rotation in and of itself eliminates both conflict-adaptation and feature-integration, however, this would predict that neither partial-repetition costs, nor conflict-adaptation should occur after rotating the boxes 360^∘^. In our third experiment, we sought to test this hypothesis.

## EXPERIMENT 3

In Experiment 3, the boxes in which stimuli appeared were rotated in similar fashion to Experiment 1, thereby again conceptually replicating two of the three rotation conditions: in one third of the trials, the boxes did not move at all (the static condition of Experiment 1 or the 0^∘^ condition of Experiment 2) and in another third of the trials, they rotated 180^∘^. Crucially for this experiment, however, was the new 360^∘^ condition in which the boxes rotated fully around their axis. Thus, if a conflict-inducing stimulus first appeared left, it would rotate to its original location. If rotating itself eliminates conflict-control, no conflict-adaptation was predicted after a 360^∘^ rotation. However, if conflict-adaptation would depend on episodic retrieval, significant conflict-adaptation should still be present.

### METHOD

Twenty students from Leiden University between the age of 18 and 27 took part in the study in exchange for course credits or money. As in Experiment 1, S1 and S2 could only appear to the left and right of the screen. Also similar to Experiment 1, the boxes in which S1 initially appeared either kept their fixed positions or gradually rotated around their axis during the ISI. Unlike the previous experiments, however, the ISI was either 800 or 1600 ms to examine whether there could be a confounding effect of rotation (in degrees) on rotation-speed (which should be important for tracking, cf. Pylyshyn and Storm, 1988). Two thirds of the trials replicated the static and rotating conditions of Experiment 1 – the boxes rotating 0^∘^ or 180^∘^ – whereas in the other third, the boxes rotated 360^∘^. The experiment lasted for approximately 50 min.

### RESULTS AND DISCUSSION

Trials with incorrect responses to S1 (10.9%) were excluded from the error analyses, and trials with incorrect responses to S1 or S2 (another 9.6%) were excluded from RT analyses.

#### Conflict-control analysis

In repeated measures ANOVAs with rotation (static vs. 180^∘^ vs. 360^∘^), ISI (800 vs. 1600 ms) and compatibility of S1 and S2, S1 compatibility had a significant effect on error proportions, *F*(1,19) = 4.50, MSe = 0.005, *p* < 0.05, but not on RTs, *F*(1,19) = 1.42, MSe = 323.01, *p* > 0.2 whereas S2 compatibility affected both RTs, *F*(1,19) = 165.86, MSe = 125054.10, *p* < 0.001, and errors, *F*(1,19) = 35.42, MSe = 0.57, *p* < 0.001. Rotation had no significant effect on RTs, *F*(2,38) = 1.91, MSe = 1578.36, *p* > 0.1 and only approached significance on errors, *F*(2,38) = 2.65, MSe = 0.02, *p* < 0.09. ISI significantly affected RTs, *F*(1,19) = 18.24, MSe = 10520.22, *p* < 0.001, and errors, *F*(1,19) = 29.28, MSe = 0.09, *p* < 0.001, with longer ISIs being 9 ms faster, but 2.7% more often incorrect. Furthermore, ISI interacted with S2 compatibility on RTs, *F*(1,19) = 6.48, MSe = 1719.27, *p* < 0.02, and errors, *F*(1,19) = 5.26, MSe = 0.04, *p* < 0.04. The effect of S2 compatibility was greater after longer ISIs (36 ms, 8.8%) than after shorter ISIs (28 ms, 5.1%). Rotation interacted with ISI on RTs, *F*(2,38) = 4.48, MSe = 1578.84, *p* < 0.02, but not on errors, *F*(2,38) = 0.61, MSe = 0.004, *p* > 0.5. Also, rotation interacted with S1 compatibility on errors, *F*(2,38) = 4.07, MSe = 0.01, *p* < 0.03, but not on RTs, *F*(2,38) = 0.85, MSe = 92.33, *p* > 0.4, and with S2 on RTs, *F*(2,38) = 12.60, MSe = 3525.34, *p* < 0.001, but not on errors, *F*(2,38) = 2.65, MSe = 0.01, *p* > 0.08.

S1 and S2 compatibility significantly interacted on RTs, *F*(1,19) = 147.53, MSe = 32287.75, *p* < 0.001 and errors, *F*(1,19) = 91.36, MSe = 0.39, *p* < 0.001. Larger S2 compatibility effects were found after compatible than incompatible S1s (50 ms and 12.6% as opposed to 16 ms and 1.2%, respectively). The three-way interaction between rotation, S1 compatibility and S2 compatibility was again significant on RTs, *F*(2,38) = 69.07, MSe = 19484.08, *p* < 0.001 and errors, *F*(2,38) = 35.69, MSe = 0.19, *p* < 0.001, showing rotating had a great effect on conflict- adaption.

**FIGURE 5:**
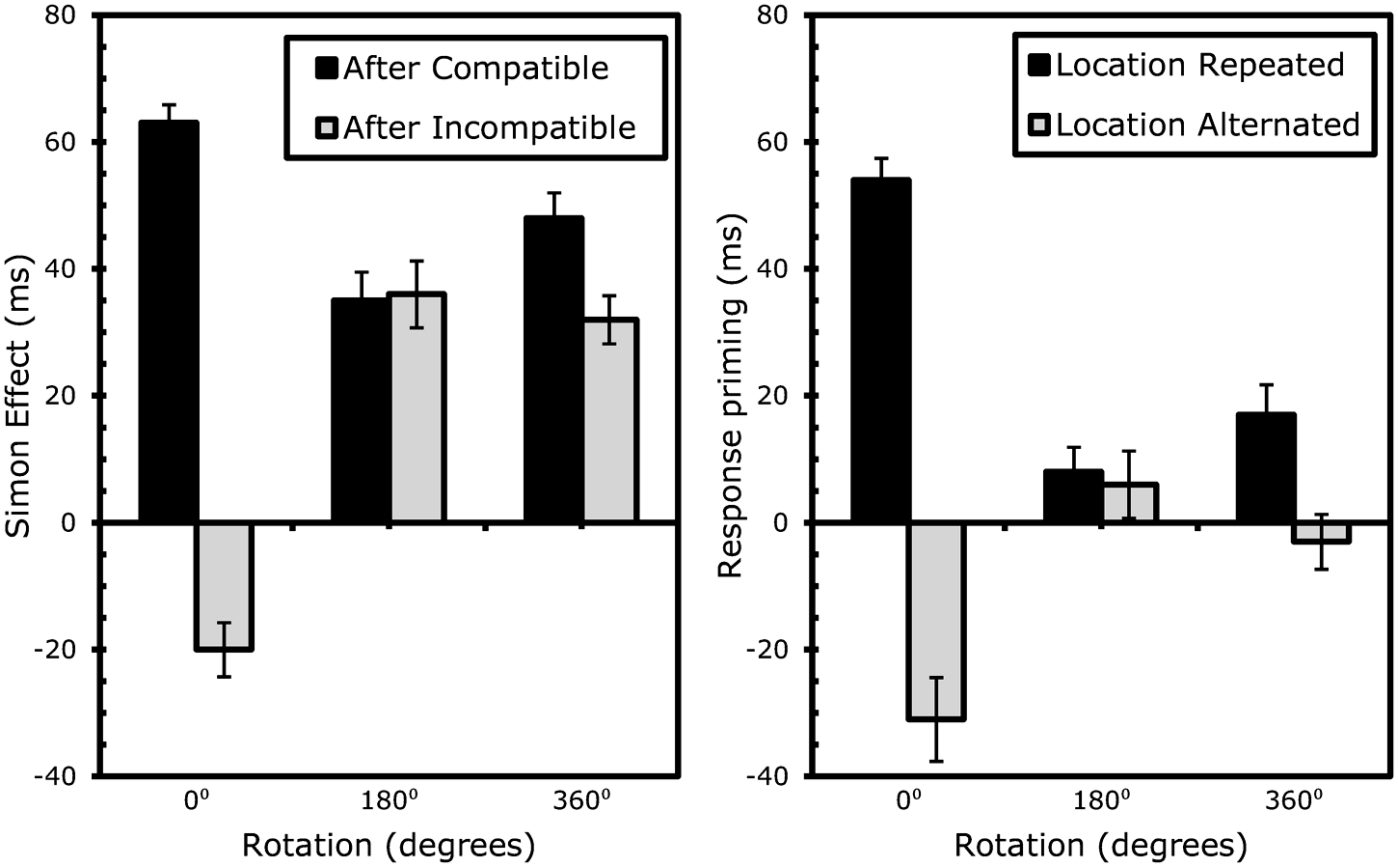
**Results Experiment 3: effects of rotation on sequential Simon effects (left) and event-coding (right).** Error bars represent standard error of the Simon (left) or response-priming (right) effect within the specific condition.

To further analyze the effects of rotation on conflict-adaptation, separate ANOVAs testing the degree to which S1 and S2 compatibility significantly interacted were conducted for each type of rotation. This interaction proved significant for static trials on RTs, *F*(1,19) = 188.91, MSe = 68587.80, *p* < 0.001, and errors, *F*(1,19) = 81.08, MSe = 0.74, *p* < 0.001. Again, after rotations of 180^∘^, the conflict adaptation pattern was completely eliminated after rotating the boxes 180^∘^ for RTs, *F*(1,19) = 0.03, MSe = 5.14, *p* > 0.8, and errors, *F*(1,19) = 0.43, MSe = 0.001, *p* > 0.5. Finally, a significant interaction was observed for trials in which the boxes rotated 360^∘^ for both RTs, *F*(1,19) = 12.57, MSe = 2662.96, *p* < 0.003, and errors, *F*(1,19) = 11.82, MSe = 0.03, *p* < 0.003. An overview of the conflict control effects is provided in **Table 3A** and **Figure 5**.

**Table 3A:** Experiment 3, compatibility and conflict-adaptation results.

					Compatibility effect		Conflict
S1	Compatible		Incompatible (i)		After c	After i	Adaptation
S2	C	I	C	I	cI – cC	iI – iC	(cI – cC) – (iI – iC)
**Reaction times**
Static	375 (7)	438 (7)	413 (8)	393 (6)	63	-20	83
180^∘^	381 (9)	416 (7)	381 (9)	417 (8)	35	36	-1
360^∘^	377 (9)	424 (8)	383 (8)	415 (9)	48	32	16
**Error rates**
Static	2 (1)	21 (2)	12 (2)	4 (1)	19	-8	27
180^∘^	3 (1)	12 (2)	4 (1)	12 (2)	9	8	1
360^∘^	3 (1)	13 (1)	5 (1)	9 (1)	10	4	6

*Reaction times, error rates and standard errors (in parentheses) for responses to S2 as a function of S2 compatibility, S1 compatibility, and rotation. Effect sizes to the right show how the compatibility effect is affected by preceding (S1) compatibility (see **Table 1A**).*

#### Event-file analysis

In repeated measures ANOVAs with rotation (static vs. 180^∘^ vs. 360^∘^), ISI (800 vs. 1600 ms), location-repetition and response-repetition, rotation had marginally significant effect on RTs, *F*(2,38) = 2.62, MSe = 2226.78, *p* < 0.09 and error proportions, *F*(2,38) = 3.11, MSe = 0.02, *p* < 0.06. Longer ISIs were significantly faster, *F*(1,19) = 22.53, MSe = 12788.14, *p* < 0.001, but also more often incorrect, *F*(1,19) = 29.68, MSe = 0.09, *p* < 0.001. Location repetition was significant for RTs, *F*(1,19) = 9.35, MSe = 1650.65, *p* < 0.01, but not for errors, *F*(1,19) = 2.85, MSe = 0.01, *p* > 0.1. Response repetition was significant for RTs, *F*(1,19) = 5.53, MSe = 9163.96, *p* < 0.03, but only marginally for errors, *F*(1,19) = 4.10, MSe = 0.02, *p* < 0.06. Repeating the response significantly interacted with ISI on RTs, *F*(1,19) = 12.89, MSe = 3474.06, *p* < 0.002, but not errors, *F*(1,19) = 0.34, MSe = 0.0004, *p* > 0.5. ISI interacted significantly with rotation on RTs, *F*(1,19) = 4.69, MSe = 1623.81, *p* < 0.02, but not on errors, *F*(1,19) = 0.62, MSe = 0.004, *p* > 0.5.

More interestingly, we replicated the overall pattern Experiment 1 and 2: location-repetition significantly interacted with response-repetition for RTs, *F*(1,19) = 178.94, MSe = 38853.61, *p* < 0.001 and errors, *F*(1,19) = 80.07, MSe = 0.46, *p* < 0.001. This interaction was modulated significantly by rotation for RTs, *F*(1,19) = 65.14, MSe = 18809.87, *p* < 0.001, and errors, *F*(1,19) = 34.41, MSe = 0.20, *p* < 0.001.

To evaluate whether the cost of partially repeating location or response was dependent on rotation, separate ANOVAs were conducted for each type of rotation (or lack thereof). For static trials, the interaction between repeating location and response was significant for RTs, *F*(1,19) = 191.64, MSe = 72363.01, *p* < 0.001 and errors, *F*(1,19) = 75.10, MSe = 0.80, *p* < 0.001, with partial-repetition costs of approximately 85 ms or 28.3%. As before, with rotations of 180^∘^, the costs were almost non-existent (2 ms or 2.3%) for RTs, *F*(1,19) = 0.37, MSe = 79.52, *p* > 0.5, or errors, *F*(1,19) = 1.55, MSe = 0.005, *p* > 0.2. However, with rotations of 360^∘^, the costs were once again clearly present (20 ms or 6.5%), for both RTs, *F*(1,19) = 19.72, MSe = 4030.82, *p* < 0.001, and errors, *F*(1,19) = 14.24, MSe = 04, *p* < 0.002. An overview of the event file analysis is provided in **Table 3B** and **Figure 5**.

**Table 3B:** Experiment 3, response-priming and Event-file results.

	Location				Response priming		Partial
	Repeated (R)		Alternated (A)		Loc. R	Loc. A	Repetition costs
Response	R	A	R	A	RA – RR	AA – AR	(RA-RR) – (AA-AR)
**Reaction times**
Static	381 (8)	436 (7)	416 (8)	386 (7)	54	-31	85
180^∘^	395 (8)	404 (9)	393 (8)	398 (9)	8	6	3
360^∘^	390 (9)	407 (9)	400 (7)	398 (9)	17	-3	20
**Error rates**
Static	1 (1)	19 (2)	14 (1)	4 (1)	18	-10	28
180^∘^	8 (1)	10 (1)	7 (1)	6 (1)	2	-1	3
360^∘^	6 (1)	8 (1)	10 (1)	6 (1)	2	-4	7

*Reaction times, error rates and standard errors (in parentheses) for responses to S2 as a function of rotation, S1-S2 location- and response-repetition. Effect sizes to the right show response-priming effects and partial-repetition costs.*

### DISCUSSION

The event-file analysis shows us that rotation in and of itself does not reduce binding cost. In Experiment 2, more degrees of rotation resulted in lower partial-repetition costs; leading to the hypothesis that rotation itself might reduce binding. Experiment 3 falsified this hypothesis: only in the 180^∘^ condition, the partial-repetition costs were completely eliminated, whereas in the 360^∘^ condition, they were again present.

More importantly, the conflict-control analysis provides evidence that rotation itself does not eliminate conflict-control. If conflict-inducing stimuli rotated back to their original location, a normal – albeit smaller – conflict adaptation pattern emerged. The previous experiments show that there is ample reason for them to be smaller. For one, if the previous location of a stimulus leaves an episodic trace of both where the box *is* and where it *had been* (as suggested by Spapé and Hommel, 2010), instances of the objects could have formed all around their axis. Since the object traveled via the opposite (180 degree) location to its former (360 or 0^∘^) place, an instance of its 180 degree position may well have been created. Second, if a participant “lost track” halfway during the rotation – i.e., paying more attention to the fact that the boxes moved as such than where they actually landed – similar patterns as during the 180^∘^ conditions would be found. This was clearly not the case. Moreover, the visually rather striking effect of rotation speed did not show clear effects on either conflict adaptation or partial repetition costs.

## GENERAL DISCUSSION

Trial-to-trial modulations in response-conflict inducing tasks are commonly taken to reflect adaptive control processes. According to this idea, conflict is registered by conflict-monitoring control process, which then signal the enhancement of the amount of control exerted (Botvinick et al., 2001; Botvinick, 2007). If so, control processes would be more efficient in trials following conflict-inducing trials, a result pattern that has been reported for various sorts of conflict tasks. In keeping with these predictions and previous observations, we were able to replicate the finding that the Simon effect is strongly reduced after incompatible trials (cf. Stürmer et al., 2002; Wühr and Ansorge, 2005). However, this sequential modulation was eliminated altogether by rather simple manipulations of the visual display in between two stimulus presentations. From a control-theoretic view this is unexpected and difficult to explain without additional assumptions, whereas an episodic approach provides a straightforward interpretation of the obtained pattern.

We have suggested that carrying out a response to a stimulus leads to the integration of stimulus and response features (shape, stimulus location, and response location in our case) into an event file that is retrieved if at least one element of the file is repeated (Hommel, 1998, 2004). Following Kahneman et al. (1992), we have assumed that visual conditions that suggest moving an object containing a stimulus to a new location induces the creation of a spatially updated file. The experiments provide evidence that this updated file also contains information about the response, so that the response in a sense travels with its object (Spapé and Hommel, 2010). The experiments also provide evidence that the updated file does not flush or overwrite the previous file, and that both files can affect performance concurrently. In the 180^∘^ conditions of all three Experiments, the impact of the two files apparently canceled each other out but when assessed separately, as in the 90^∘^ condition of Experiment 2, both could be shown to have an effect.

What do our findings imply for the relationship between adaptive control mechanisms and episodic integration and retrieval effects? We think that two different answers to this question are possible and that it would be premature to decide between them at this point. The radical response would be to consider that all effects that have been assumed to reflect adaptive control mechanisms are artifacts of priming and integration processes (cf. Schmidt, 2013). Indeed, there are more possible effects of that sort than proponents of control approaches have considered, ranging from simple repetition priming (Mayr et al., 2003) over feature integration and the partial-repetition costs they produce (Hommel et al., 2004) to contingency learning (Schmidt and De Houwer, 2011; Mordkoff, 2012) and effects of integrated competition (Duncan, 1996; Dutzi and Hommel, 2009). Even though the basic characteristics of these types of processes are reasonably well understood, it is entirely unclear how they affect performance in the standard conflict tasks and the often rather complicated task versions that have been designed to minimize episodic effects. With respect to the present study, it is fair to say that our event-coding analyses are much easier and straightforward to interpret than the conflict-control analyses, but, more importantly, that the latter are actually not needed to understand the data patterns we obtained. Thus, one might consider the reasoning underlying the conflict-control analyses as unnecessary theoretical overhead.

An alternative, less radical response could consider that adaptive control does take place and can indeed affect subsequent performance, but that the adaptations achieved by the respective control processes are entirely integrated and thus rely on episodic event files (for a somewhat similar suggestion, see Verguts and Notebaert, 2009). For instance, a given file may not only contain pointers to, or associations with codes of stimulus and response features but also information about association weights, that is, about how strongly each given stimulus feature is associated with, or predicts successful responses. There are several observations that are consistent with this scenario. For one, it has been observed that event files are relatively liberal with regard to the precise timing relation between the stimuli and the responses they integrate, as long as the stimuli appear close to response execution (Hommel, 2005). This might suggest that the integration takes place vis-à-vis an evaluation of the response’s success and is informed by the outcome of this evaluation.

Consistent with that possibility is the observation that the partial-repetition costs that we attribute to event files are systematically affected by experimental manipulations impacting the current dopamine level: Partial-repetition costs are positively correlated with the spontaneous eye-blink rate, a marker of dopaminergic activity (Colzato et al., 2007a); they increase if stimulus-response pairings are followed by task-irrelevant pictures with positive affective content (Colzato et al., 2007b), stimuli that are suspected to induce a phasic increase of the individual dopamine level (Ashby and Isen, 1999; Cohen et al., 2002); and they decrease in the case of stress, a condition that is known to induce an overproduction of dopamine (Colzato et al., 2008). Given the evidence that phasic changes in the dopamine level are essential for success-controlled learning and stimulus-response integration (Schultz, 2002), these findings fit with the idea that the creation of event files is regulated by success. If we further assume that success triggers the integration of information about all processing aspects that were responsible for it and consider that the cognitive states underlying the efficient handling of response conflicts belong to those aspects, it makes sense to think that event files include control-relevant information. If so, some part of trial-to-trial modulations in conflict tasks may well reflect adaptive control processes and finding that these modulations are in a sense controlled by episodic retrieval does not necessarily imply a contradiction. This idea fits well with later revisions of the conflict-monitoring hypothesis (Botvinick, 2007) that consider conflict as aversive stimuli that operate as teaching signals to avoid using the same selection of associated tasks and strategies. Accordingly, after conflict trials that are accompanied by rewarding stimuli, conflict adaptation is reduced (Van Steenbergen et al., 2009). Indeed, a more adaptive form of cognitive control operation would apply control-relevant information in comparable situations only – that is, in situations that trigger the retrieval of episodic memories related to that situation.

Although this interpretation would be in line with the present results, current theorizing seems to restrict itself to the boundaries of either conflict-control or event-files while their possible interdependency is left to be accounted for. In contrast to Akçay and Hazeltine (2008) or Spapé and Hommel (2008), who found conflict-adaptation to be dependent on the context of the stimulus or the task, others (e.g., Freitas et al., 2007) still found conflict-adaptation even when task-relevant features changed between trials, making the present state of affairs heterogeneous. Rather than arguing that the effects of sequential conflict effects are a by-product of pure stimulus/response-repetition or feature-integration as such, we feel that a framework that focuses on the interplay of control and episodic retrieval could provide the more adequate solution to this puzzle. One of the greater challenges, then, becomes to be able to predict which contextual discontinuities reduce episodic retrieval, thereby disrupting or preventing cognitive control and adaptation. The presented experiments provide several examples of such episodic boundaries of control, and we hope they will inspire future research to focus on re-integrating the fields of executive control with episodic memory.

To conclude, our findings suggest that sequential modulations of conflict effects, the bread-and-butter of adaptive-control approaches, are strongly dependent on episodic retrieval and disappear under conditions that make episodic retrieval unlikely. At a minimum, the findings add to the evidence that demonstrate that sequential modulations cannot be taken to represent process-pure measures of adaptive control (c.f. Hommel et al., 2004; Risko et al., 2008). Possibly, all presented effects may be accounted for entirely in terms of episodic effects. Alternatively, an intriguing compromise could be that control-relevant information is integrated into event files and retrieved only if the current situation is sufficiently similar to the situation in which the event file was originally created.

## Conflict of Interest Statement

The authors declare that the research was conducted in the absence of any commercial or financial relationships that could be construed as a potential conflict of interest.
